# The Unique Mechanisms of Cellular Proliferation, Migration and Apoptosis are Regulated through Oocyte Maturational Development—A Complete Transcriptomic and Histochemical Study

**DOI:** 10.3390/ijms20010084

**Published:** 2018-12-26

**Authors:** Błażej Chermuła, Maciej Brązert, Michal Jeseta, Katarzyna Ożegowska, Patrycja Sujka-Kordowska, Aneta Konwerska, Artur Bryja, Wiesława Kranc, Maurycy Jankowski, Mariusz J. Nawrocki, Ievgeniia Kocherova, Piotr Celichowski, Blanka Borowiec, Małgorzata Popis, Joanna Budna-Tukan, Paweł Antosik, Dorota Bukowska, Klaus P. Brussow, Leszek Pawelczyk, Małgorzata Bruska, Maciej Zabel, Michał Nowicki, Bartosz Kempisty

**Affiliations:** 1 Division of Infertility and Reproductive Endocrinology, Department of Gynecology, Obstetrics and Gynecological Oncology, Poznan University of Medical Sciences, 60-535 Poznań, Poland; blazej.chermula@wp.pl (B.C.); maciejbrazert@ump.edu.pl (M.B.); katarzyna.ozegowska@ump.edu.pl (K.O.); pawelczyk.leszek@ump.edu.pl (L.P.); 2 Department of Obstetrics and Gynecology, University Hospital and Masaryk University, 601 77 Brno, Czech Republic; jeseta@gmail.com; 3 Department of Histology and Embryology, Poznan University of Medical Sciences, 60-781 Poznań, Poland; psujka@ump.edu.pl (P.S.-K.); akonwer@ump.edu.pl (A.K.); pcelichowski@ump.edu.pl (P.C.); jbudna@ump.edu.pl (J.B.-T.); mnowicki@ump.edu.pl (M.N.); 4 Department of Anatomy, Poznan University of Medical Sciences, 60-781 Poznań, Poland; abryja@ump.edu.pl (A.B.); wkranc@ump.edu.pl (W.K.); m.jankowski.14@aberdeen.ac.uk (M.J.); mjnawrocki@ump.edu.pl (M.J.N.); ikocherova@ump.edu.pl (I.K.); blanka.maria.b@gmail.com (B.B.); malgorzatapopis111@gmail.com (M.P.); mbruska@ump.edu.pl (M.B.); 5 Veterinary Center, Nicolaus Copernicus University in Torun, 87-100 Toruń, Poland; pantosik@umk.pl (P.A.); dbukowska@umk.pl (D.B.); prof.bruessow@gmail.com (K.P.B.); 6 Department of Histology and Embryology, Wroclaw University of Medical Sciences, 50-368 Wrocław, Poland; mazab@ump.edu.pl; 7 Division of Anatomy and Histology, University of Zielona Gora, 65-046 Zielona Góra, Poland

**Keywords:** pig, oocytes, microarray, cellular competence

## Abstract

The growth and development of oocyte affect the functional activities of the surrounding somatic cells. These cells are regulated by various types of hormones, proteins, metabolites, and regulatory molecules through gap communication, ultimately leading to the development and maturation of oocytes. The close association between somatic cells and oocytes, which together form the cumulus-oocyte complexes (COCs), and their bi-directional communication are crucial for the acquisition of developmental competences by the oocyte. In this study, oocytes were extracted from the ovaries obtained from crossbred landrace gilts and subjected to in vitro maturation. RNA isolated from those oocytes was used for the subsequent microarray analysis. The data obtained shows, for the first time, variable levels of gene expression (fold changes higher than |2| and adjusted *p*-value < 0.05) belonging to four ontological groups: regulation of cell proliferation (GO:0042127), regulation of cell migration (GO:0030334), and regulation of programmed cell death (GO:0043067) that can be used together as proliferation, migration or apoptosis markers. We have identified several genes of porcine oocytes (*ID2*, *VEGFA*, *BTG2*, *ESR1*, *CCND2*, *EDNRA*, *ANGPTL4*, *TGFBR3*, *GJA1*, *LAMA2*, *KIT*, *TPM1*, *VCP*, *GRID2*, *MEF2C*, *RPS3A*, *PLD1*, *BTG3*, *CD47*, *MITF*), whose expression after in vitro maturation (IVM) is downregulated with different degrees. Our results may be helpful in further elucidating the molecular basis and functional significance of a number of gene markers associated with the processes of migration, proliferation and angiogenesis occurring in COCs.

## 1. Introduction

The formation of fully mature oocyte consists of nuclear and cytoplasmic events, which are necessary for the acquisition of the competence for their fertilization, and further development of the embryo. Development of oocytes begins in the fetal ovary, whereas the final oocyte growth and maturation occurs during adulthood [1]. During this long meiotic arrest, the oocyte increase in size by the acquisition of maternal transcriptions and proteins [2].

The growth and development of oocyte affect the functional activities of the surrounding somatic cells. These cells are regulated by various types of hormones, proteins, metabolites and regulatory molecules through gap communication, ultimately leading to the development and maturation of oocytes [3]. The close association between somatic cells and oocytes, which together form the cumulus-oocyte complexes (COCs), and their bi-directional communication are crucial for the acquisition of developmental competences by the oocyte.

Growing oocytes in the ovary acquire the ability to mature through a two-way communication between the gamete and surrounding somatic cumulus cells (CCs) [4,5,6]. Oocyte secretes many growth factors that promote differentiation and proliferation of CCs [7]. These molecules are mostly responsible for the maintenance of the diverse state of CCs, preventing them from differentiating into theca or granulosa cells [8]. The replacement of molecules is carried out through gap junctions which connect oocyte membrane and the CCs. One of the most important transported macromolecules are nucleic acids, such as RNA [9]. They can be transported bi-directionally, however, specific functions and transport mechanism are still not explained. Genetic and transcriptional profile of COCs reflects in the developmental potential for successful fertilization and embryo development. However, little is known about the relationship between genes expressed in oocytes and cumulus cells [10].

Microarray DNA technology is a useful method to examine changes in gene expression. Analysis of the oocyte transcriptome could be a powerful tool for improving our knowledge about the pathways involved in oocyte development [11]. Microarray technology has enabled the discovery of many ontological groups, closely or loosely associated with oocyte development or CC proliferation and apoptosis [12].

In this study, the aim was to identify new gene markers involved in cellular proliferation, migration and apoptosis. In vitro cultures of porcine COCs were performed. Then the transcriptomic profile changes of oocyte genes involved in regulation of cell migration, cell proliferation, and programmed cell death, was investigated. The genes whose expression values differed significantly in porcine oocytes, when analyzed before (immature) and/or after in vitro maturation (IVM; matured), were analyzed. Our results could provide evidence about the processes of communication between oocyte and cumulus cells and allow to define the genes responsible for the functioning of CCs, which are directly related with oocytes.

## 2. Results

Whole transcriptome profiling using Affymetrix microarray allowed us to analyze the gene expression changes in freshly isolated oocytes, before in vitro procedure (“Before IVM”), in relation to after in vitro maturation (“After IVM”). Using Affymetrix® Porcine Gene 1.1 ST Array we have detected the expression of 12,258 porcine transcripts. To be taken under consideration as differentially expressed, the genes needed to express a fold change higher than |2| and with a corrected *p*-value < 0.05. Applying these criteria yielded 419 different genes. This group was subjected to significantly enriched GO BP identification. All of the raw microarray results were uploaded to the GEO database (available at: https://www.ncbi.nlm.nih.gov/geo/query/acc.cgi?acc=GSE97246).

**Figure 1:**
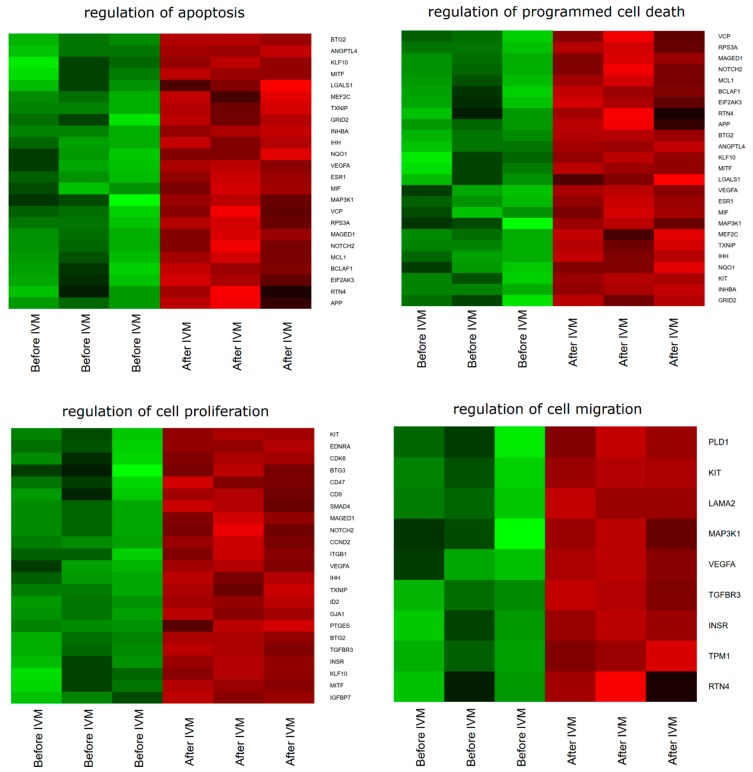
Heat map representations of differentially expressed genes belonging to the “cell proliferation”, “regulation of programmed cell death”, and “regulation of cell migration” GO BP (gene ontology Biological Process) terms. Arbitrary signal intensity acquired from microarray analysis is represented by colors (green, higher; red, lower expression). Log2 signal intensity values for any single gene were resized to Row Z-Score scale (from −2, the lowest expression to +2, the highest expression for the single gene, each). Each copy of the analyzed groups represents a different biological sample.

DAVID (Database for Annotation, Visualization and Integrated Discovery; https://david.ncifcrf.gov/) software was used for extraction of the genes belonging to the “regulation of cell proliferation”, “regulation of programmed cell death”, and “regulation of cell migration” gene ontology Biological Process terms (GO BPs). We found that 42 genes from these GO BP terms were significantly represented in down-regulated gene sets. These sets of genes were subjected to hierarchical clusterization procedure and presented as heat maps (Figure 1).

Set of the differentially expressed genes belonging to “cell proliferation”, “regulation of programmed cell death”, and “regulation of cell migration” GO BP terms with their official gene symbols, fold changes in expression and corrected p values were shown in Table 1.

**Figure 2:**
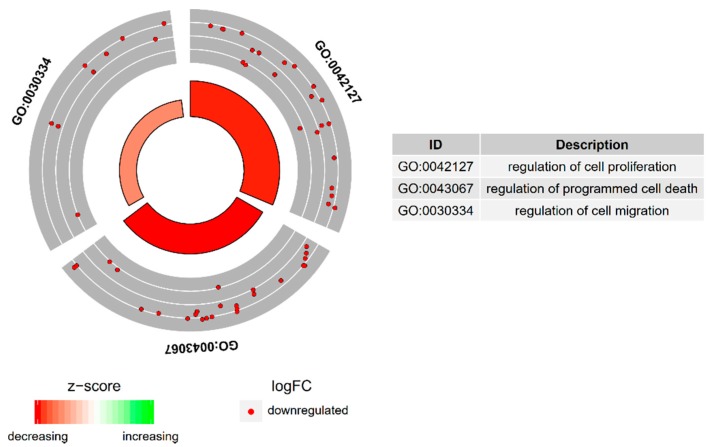
The circle plot showing the differently expressed genes and z-score of “cell proliferation”, “regulation of programmed cell death”, and “regulation of cell migration” GOs. The outer circle shows a scatter plot for each term of the fold change of the assigned genes. Red dots display down-regulation. The inner circle shows the z-score of each GO BP term. The width of each bar corresponds to the number of genes within GO BP term and the color corresponds to the z-score.

The enrichment of each GO BP term as well KEGG pathway were calculated as z-score and shown on the circle diagram (Figure 2).

**Figure 3:**
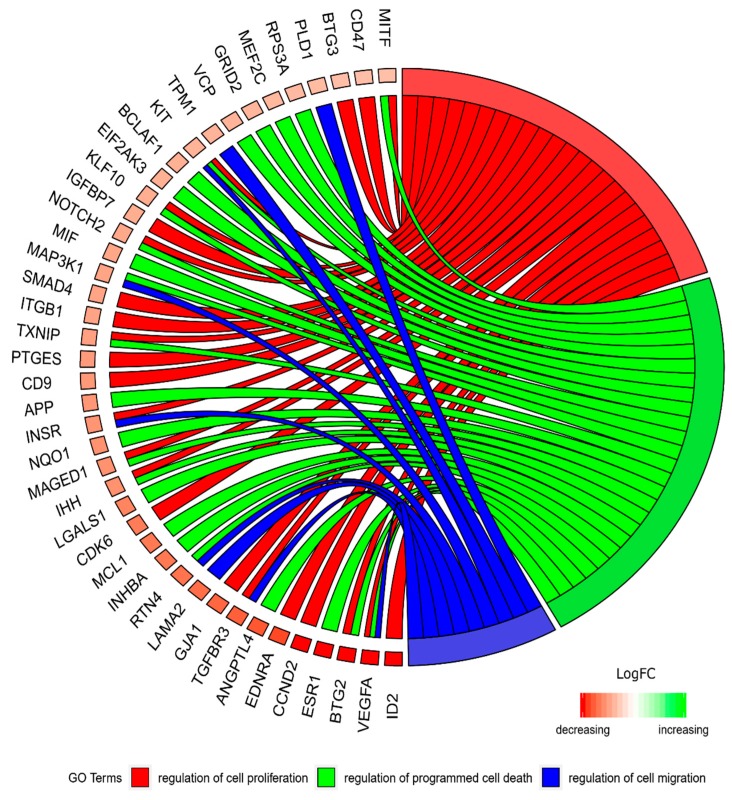
The representation of the mutual relationship between differentially expressed genes that belongs to the “cell proliferation”, “regulation of programmed cell death”, and “regulation of cell migration”. The ribbons indicate which gene belongs to which categories. The middle circle represents logarithm from fold change (LogFC) between D7/D1, D15/D1 and D30/D1 respectively. The genes were sorted by LogFC from the most to the least changed gene.

**Figure 4:**
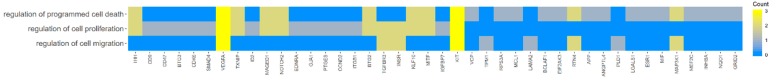
Heatmap showing the gene occurrence between differentially expressed genes that belong to the “cell proliferation”, “regulation of programmed cell death”, and “regulation of cell migration”. The red color is associated with gene occurrence in the GO Term. The intensity of the color is corresponding to the amount of GO BP terms that each gene belongs to.

Moreover, the genes that are part of a particular GO, can at the same time be members of different gene ontologies. This prompts for an investigation of the relationships between the genes of interested and the respective GOs. The relation between those GO BP terms was presented as a circle plot (Figure 3) as well as a heatmap (Figure 4).

**Figure 5:**
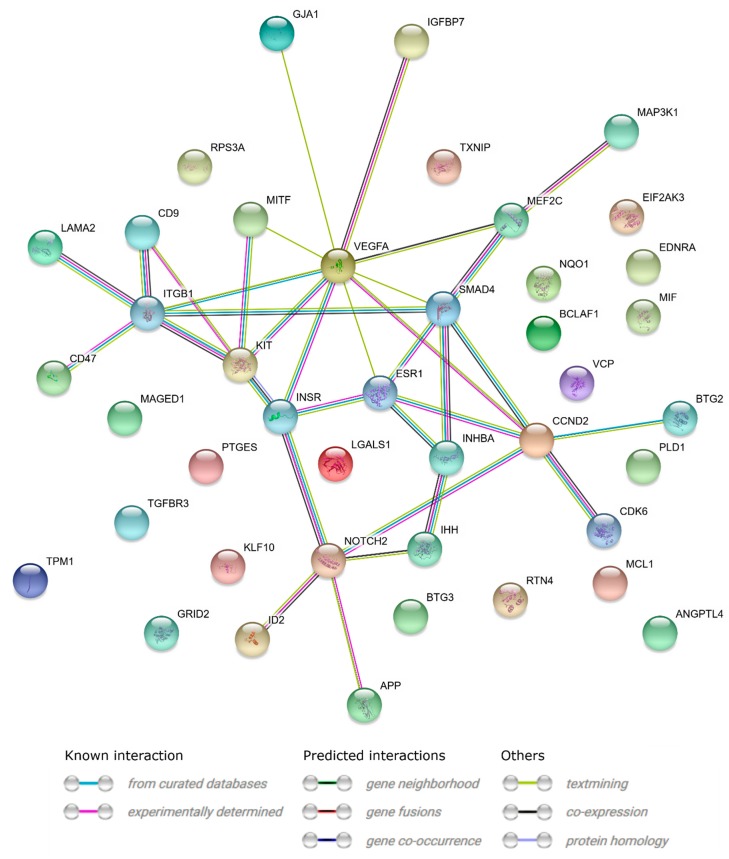
STRING-generated interaction network between genes that belongs to the “cell proliferation”, “regulation of programmed cell death”, and “regulation of cell migration”. The intensity of the edges reflects the strength of the interaction score.

STRING-generated interaction network was created between differentially expressed genes belonging to the analyzed ontology groups. The intensity of the edges reflects the strength of the interaction score (Figure 5).

**Figure 6:**
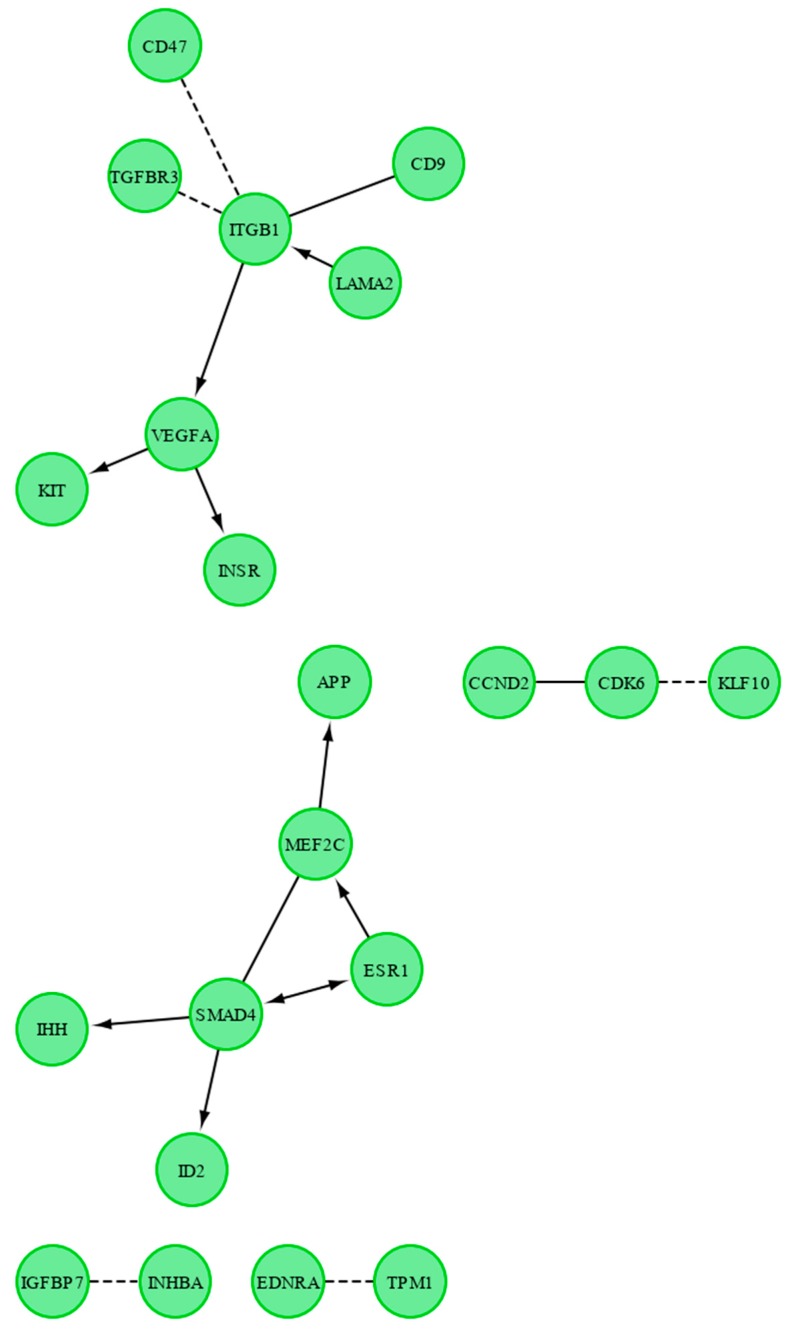
Functional interaction (FI) between the analyzed downregulated genes that belong to the “cell proliferation”, “regulation of programmed cell death”, and “regulation of cell migration”. In the following figure “->“ stands for activating/catalyzing, “-|” for inhibition, “-” for FIs extracted from complexes or inputs, and “---” for predicted FIs.

Finally, we investigated the functional interactions between chosen genes with REACTOME FIViz app to Cytoscape 3.6.0 software (https://cytoscape.org/; http://apps.cytoscape.org/apps/reactomefiplugin). The results are shown in Figure 6.

**Figure 7:**
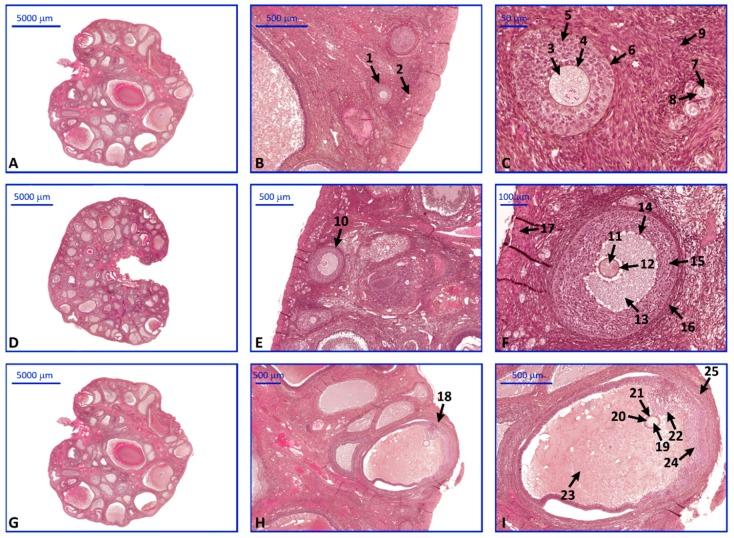
Representative sections of pubertal crossbred Landrace gilt ovaries stained with (**H**) and (**E**). (**A**–**C**). primordial and primary follicles, (**D**–**F**). secondary follicle, (**G**–**I**). Graafian follicle. Arrows: 1—primary follicle, 2—primordial follicle, 3,7,11—primary oocyte, 4,12,20—zona pellucida, 5,14,24—granulosa cells, 6,15—theca cells, 8—follicular cells, 9—cortical stroma, 10—secondary follicle, 13,23—antrum, 16,25—theca externa, 17—tunica albuginea, 18—Graafian follicle, 19—secondary oocyte, 21—corona radiata, 22—cumulus oophorus.

Light microscope observations of collected tissues revealed a normal ovarian morphology. In each of the analyzed ovaries, follicles in various stages of growth were present (Figure 7).

**Figure 8:**
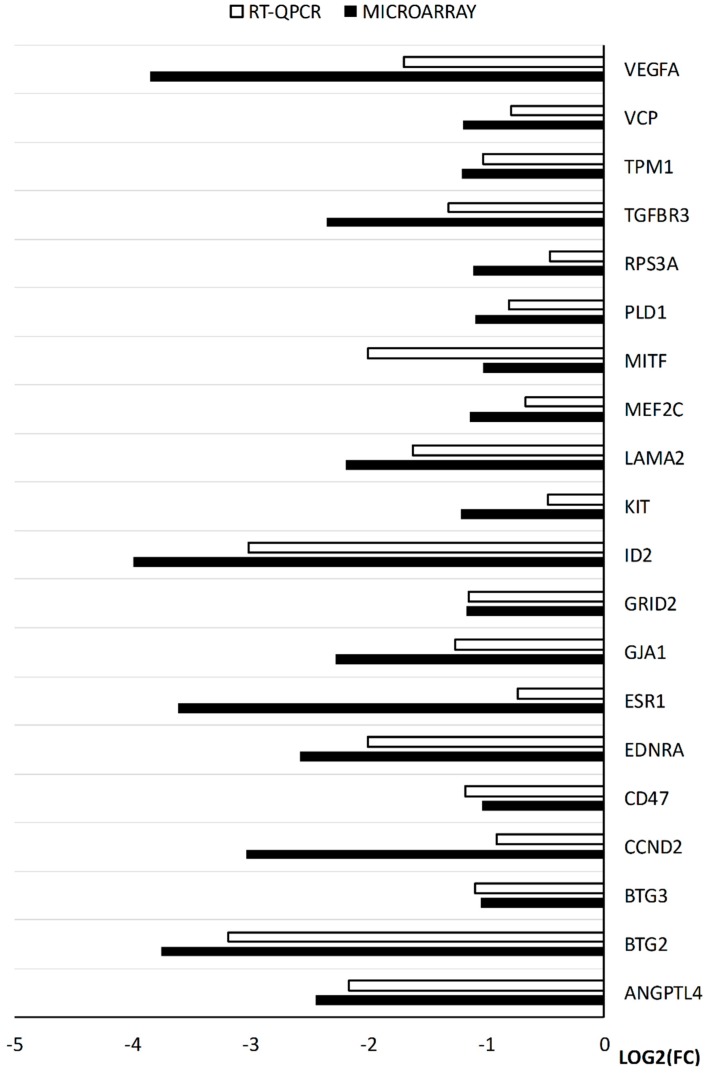
The results of the RT-qPCR validation of the analyzed genes, presented in a form of bar graph.

The RT-qPCR analysis presented results comparable to those obtained with the use of microarrays. In every example, the direction of changes was confirmed. In the same time, the scale of changes often varies significantly between the methods. However, this difference is not surprising, considering the different accuracy of complete transcriptomic analysis and a specific primer based, single gene oriented approach. In the end, the RT-qPCR is far more accurate in quantitative analysis of transcript levels, as it targets specific gene sequences as opposed to multiple probes for different transcript variants of the same gene. The results were compiled into a bar graph and presented as Figure 8.

## 3. Discussion

Development of oocytes is supported by somatic cells such as granulosa and theca cells. In the mature follicle there are two separate populations of granulosa cells; mural granulosa cells (GCc) and cumulus cells (CCs). CCs are directly related to the oocyte forming cumulus-oocyte complexes (COCs) together [13]. GCs play an important role in the process of folliculogenesis and ovarian follicle development [14]. The communication that exists between the oocyte and granulosa cells is important for the oocyte development and maturation [15]. Denudation of oocytes can cause a strong effect on meiotic maturation which is not evident in morphological aspects but can make big differences in organelle distribution, gene expression or protein synthesis. Oocytes are connected with the accompanying cells through gap junctions which represent the key communication pathways that control the development of oocytes in ovarian follicles [16]. Effective communication between CCs and oocytes is important for correct oocyte development but is not necessary for meiotic maturation to second metaphase stage.

Recognition of interactions in a microenvironment of COCs is the key to understanding the physiology process at this system level. It is important to learn how the molecular interactions between the oocyte and CCs proceed. Acquisition of full developmental ability for fertilization by oocytes is accompanied by morphological and biochemical changes. These modifications occur during long stages of folliculogenesis and oogenesis in both oocytes and CCs [17,18]. There is growing evidence that CCs play a key role in folliculogenesis and oocyte development to the acquisition of fertilization competence [19]. The development of ovaries and ovarian follicles requires proper expression of various genes at every stage of development.

Molecular interactions and their complexity between the oocyte and the environment CCs during folliculogenesis are not well known. A new method of gene expression analysis such as microarrays enabled to discover a new look at COC functions and pathways activated during follicular development. The microarray technique provides a global analysis of the tissue transcriptome and is a hypothesis-generating tool [20]. Our study investigates gene expression in porcine oocytes in order to define differentially expressed genes belonging to the ontology groups associated with the regulation of programmed cell death, cell proliferation and cell migration.

Microarray gene expression analysis was performed on immature and in vitro matured porcine oocytes The obtained data points to a key role of genes belonging to this functional groups. Furthermore, it is suggested that the expression of these genes can play a positive role in the future embryo development. Our study investigates how the expression of genes responsible for migration, proliferation or apoptosis changes during oocyte maturation. Considering the close interaction and type of communication between oocyte and cumulus cells, the expression of genes belonging to these four groups may have a direct impact on the occurrence of these processes in the somatic cell population directly related to the oocyte membrane.

The relationship between transcription levels of expressed genes in oocytes and cumulus cells strongly indicates that the interaction between oocyte and CCs is partly modulated by the mechanisms of gene regulation [10]. It also seems interesting to determine whether the analyzed mRNA comes from expressed genes or is a residual transcriptome from the early stages of oogenesis. During folliculogenesis, oocyte accumulates a rich diversity of transcripts and protein. Co-expression analysis in oocytes has shown that there is a coordinated regulatory mechanism that drives the transcription and accumulation of 2222 gene products [21].

Despite above reports, there are not many cases regarding the transcriptomic profile analysis of immature and matured pig oocytes. Those available that have brought these topics closer to understanding, pointed out a new look at functions of the detected genes during maturation of pig oocytes [12]. In our research, we have paid particular attention to the study of the expression of genes involved in the processes of proliferation, migration and apoptosis and programmed cell death occurring in COCs. The basis of this assumption is the fact that oocytes regulate gene expression in CCs and vice versa. Results of experiments conducted by Biase et al. present that the transcripts of several hundred genes expressed in oocytes show a linear correlation with genes expressed in CCs. Moreover, the functional organization of a select group of co-expressed genes exist [10].

All four analyzed ontological groups show the most important processes for the maturing oocyte beginning from the early stages of folliculogenesis to form COCs and achieve nuclear maturity. CCs proliferation and COCs differentiation are thought to define the developmental competence of COCs for growth and development, as well as further embryonic viability [22]. Studies by Kempisty et al. clearly showed that oocyte-cumulus communication supports the proliferation and in vitro differentiation of somatic cells [23]. Transcript analysis of CCs derived from oocytes with high developmental capacity allowed to observe the arrest of the proliferation process, and an increase in apoptotic activity [24,25]. This hypothesis is supported by another study that shows apoptotic growth activity in CCs from MII oocytes as compared to GV oocytes [26].

In our research, using a microarray approach, we wanted to study the porcine oocyte transcriptomic profile before and after in vitro maturation (IVM) to define new molecular markers in female gametes associated with their ability to mature. It was found that all of the 42 analyzed genes belonging to the analyzed ontology groups are downregulated after IVM. From those differentially expressed genes we have selected and analyzed *ID2*, *VEGFA*, *BTG2*, *ESR1*, *CCND2*, *EDNRA*, *ANGPTL4*, *TGFBR3*, *GJA1*, *LAMA2* genes which are most downregulated and group of *KIT*, *TPM1*, *VCP*, *GRID2*, *MEF2C*, *RPS3A*, *PLD1*, *BTG3*, *CD47*, *MITF* genes which expression is less downregulated after IVM culture.

The most downregulated gene that belongs only to the “regulation of cell proliferation” ontology group is *ID2* (inhibitor of DNA binding 2). This gene encodes a protein belonging to a DNA-binding family of inhibitors whose members are transcriptional regulators, exhibiting non-primary helix-loop-helix (HLH) domains. The protein encoded by *ID2* may play a role in the negative regulation of cell differentiation [27]. Level of this gene’s expression remains in line with early reports by Budna et al. [28]. *ID2* expression is regulated in response to countless stresses, including hypoxia and ischemia, suggesting a role in adaptive cellular responses to metabolic stress [29]. A definite reduction in the expression of this gene in the maturing oocyte may indicate its role in reaching maturity and further fertilization. The second most downregulated gene that belongs to all four analyzed ontology groups is *VEGFA* (vascular endothelial growth factor A). This growth factor induces proliferation and migration of vascular endothelial cells and is upregulated in many known cancers. Its expression correlates with the degree of cancer progression. *VEGFA* is the main factor and regulator of angiogenesis in the ovulatory follicle and promotes endothelial cell migration [30,31,32]. In vitro maturation of bovine oocytes with VEGF supplementation, results in improving the cytoplasmic maturation and ability for oocyte development [33,34]. We found interactions between *VEGFA* and *KIT* (proto-oncogene receptor tyrosine kinase) or *INSR* (insulin receptor) genes. *VEGFA* activates *KIT* and *INSR*, which are mainly related to the positive regulation of cell proliferation and migration. Activities of this three genes can be regulated by the porcine oocyte maturation process, participating together in differentiation and development of COCs [22].

*BTG2* (B cell translocation gene 2) belongs to all analyzed ontology groups. *BTG2* has anti-proliferative properties and belongs to the family of genes that are involved in development, death, apoptosis, differentiation and survival of cells [35]. Next of the most downregulated genes related to the regulation of programmed cell death and apoptosis GOs are *ESR1* (estrogen receptor 1) and *ANGPTL4* (angiopoietin-like 4). *ESR1* gene is involved in the ovarian response to exogenous FSH. Since the first reports about estrogens effects on the ovarian follicle growth, maturation, and release of the oocyte, *ESR1* has been associated as a marker [36]. In turn, angiopoietin-like 4 also acts as an apoptosis survival factor for vascular endothelial cells and may prevent metastasis by inhibiting the growth of blood vessels. *ANGPTL4* is characterized as a gene associated with angiogenesis, it plays a key role in the late stages of folliculogenesis, taking part in providing nutrients and oxygen to growing follicles [37]. Expression of this gene is mostly associated with pathologies and caused by microenvironment factors, such as hypoxia, which may explain the decline in its expression during oocyte IVM. Other downregulated genes are *CCND2* (cyclin D2), *EDNRA* (endothelin receptor type A) and *GJA1* (gap junction protein alpha 1). They all belong to the “regulation of cell proliferation” ontology group. Homologous knockout studies in mice suggest the key role of *CCND2* in ovarian GCs and germ cell proliferation. Cyclin D2 mRNA is presented in GCs of growing vesicles. Their expression was induced by FSH and quickly inhibited by LH surge [38]. Presence of *CCND2* in human CCs during maturation of oocytes is a marker of poor prognosis for embryo development and consequently lower fertility [39]. *EDNRA* is responsible for the production of endothelin type A receptor and regulates muscle cells vasoconstriction. EDNRA protein level is significantly reduced in the uteroplacental vascular bed [40]. Microarray results from in vitro and in vivo models presented by Kawamura et al. show that endothelin-1 acting with EDNRA could promote germinal vesicle breakdown in preovulatory oocytes [41]. This may explain the lowered expression of this gene in oocytes after IVM. *GJA1* belongs to connexin gene family that encodes a protein which is a part of gap junction communication channels, allowing the diffusion of low-mass molecules from cell to cell. *GJA1*, also known as *connexin 43*, is mainly expressed in GCs, participating in connexons with other GCs or with the oocyte through CCs. *Connexin 43* was identified as a gene marker for oocyte and embryo quality [42]. *GJA1* regulates communication between oocyte and CCs through its involvement in gap junctions. Lower *GJA1* levels are favourable for oocyte maturation. Sheng-Hsiang proved that lower expression of *GJA1* is observed in CCs obtained from matured oocytes compared to immature ones [43]. This discovery reflects our results in the case of matured oocytes. Last two out of ten most downregulated selected genes are *TGFBR3* (transforming growth factor β receptor 3) and *LAMA2* (laminin subunit alpha 2). *TGFBR3* is important in the development of coronary arteries and various tissue blood vessels. In cancers, reduced expression of this receptor has been observed [44]. Elevated expression of TGFBR3 was observed in the CC population exposed to temporary hypoxia [37]. Thus, elevated expansions CCs is in contradiction with the lowering gene expression in oocytes after IVM. This may be due to the fact that oocyte itself does not participate in the formation of the corpus luteum blood vessels after ovulation. Belonging to the “regulation of cell migration” ontology group, *LAMA2* gene activates *ITGB1* (integrin subunit β 1) and encodes the alpha-2 chain of laminin-2. It was suggested that *LAMA2* or *TPM1* (tropomyosin 1) encode proteins involved in the formation of oocyte microtubule structure and may affect to the ability of porcine oocyte for maturation. *TPM1*, which belongs to the group of less downregulated genes can be important in connection and reorganization of the actin microfiber complex during COC maturation. The protein encoded by this gene probably plays a key role in reaching oocyte cellular maturation [22]. The gene whose expression changes in the slightest degree after oocyte IVM is *MITF* (melanogenesis associated transcription factor). *MITF* belongs to all the analyzed ontology groups except “regulation of cell migration” and has been connected with osteogenesis [45]. The role of this gene in the maturation of oocytes has not been investigated so far. Only Celichowski et al. demonstrated that this gene is upregulated before IVM [34]. *CD47* (CD47 molecule) and *BTG3* (BTG anti-proliferation factor 3) represent the group of genes which are only related to the regulation of proliferation. *CD47* encodes a membrane protein playing an important role in transmembrane transport and signal transduction. *CD47* gene is upregulated in tumours that inhibit innate immune responses, including macrophage phagocytosis [46]. In turn, the BTG3 protein appears to have antiproliferative properties and might play a role in neurogenesis. It was identified as a candidate gene responsible for various cancers. BTG family members can regulate granulosa cell differentiation into lutein cells [47]. *PLD1* (phospholipase D1) participates in signal transduction. Together with *PLD2* (phospholipase D1), it is expressed in a wide variety of tissues and cells. A small part of PLD1 is located in the plasma membrane, where it contributes to mediating in certain signalling events [48]. Last four genes classified to the least downregulated group are *RPS3A* (ribosomal protein S3A), *MEF2C* (myocyte enhancer factor 2C), *GRID2* (glutamate ionotropic receptor delta type subunit 2) and *VCP* (valosin-containing protein). These genes are characterized by a very similar level of expression and belong to the programmed cell death ontology group. Expression of ribosomal protein S3A was decreased in human EAT (epicardial adipose tissue) [49]. *GRID2* is a member of ionotropic glutamate receptor family that mediates in synaptic transmission [50]. *VCP* was identified as one of the necessary genes in 25 ovarian cancer cell lines, as well as 75 non-ovarian cell lines [51]. So far no direct relationship between processes occurring in ovarian tissue caused by the last four analyzed genes has been reported. However, their expression could be important for determining new biomarkers for oocyte maturation.

In summary, we have identified several genes of porcine oocytes, whose expression after IVM is downregulated with different degrees. This data shows, for the first time, variable levels of gene expression belonging to four ontological groups: regulation of cell proliferation (GO:0042127), regulation of cell migration (GO:0030334), and regulation of programmed cell death (GO:0043067), that can be used together as proliferation, migration or apoptosis markers. What is more, all analyzed genes have been significantly downregulated after IVM compared to oocytes assessed before IVM. Our findings show that most of the genes expressed in oocytes are co-expressed and functionally depended on cumulus cells. Close communication between oocyte and CCs, conducted in two directions by a number of mediators and membrane receptors, is a decisive factor in the expression of oocyte genes. Our previous and current results show that the interaction that occurs between oocyte and CCs is not just an exchange of metabolites, it is mainly related to the expression of the same genes on both sides. It is interesting to note that significant expression reduction after IVM occurs, mainly involving genes that were directly or indirectly related to the functioning of the oocytes themselves as well as with CCs. These genes which are less downregulated are not specific to the oocyte itself. Our results may be helpful in further elucidating the molecular basis and functional significance of a number of gene markers associated with the processes of migration, proliferation and angiogenesis occurring in COCs.

## 4. Material and Methods

### 4.1. Experimental Design

**Figure 9:**
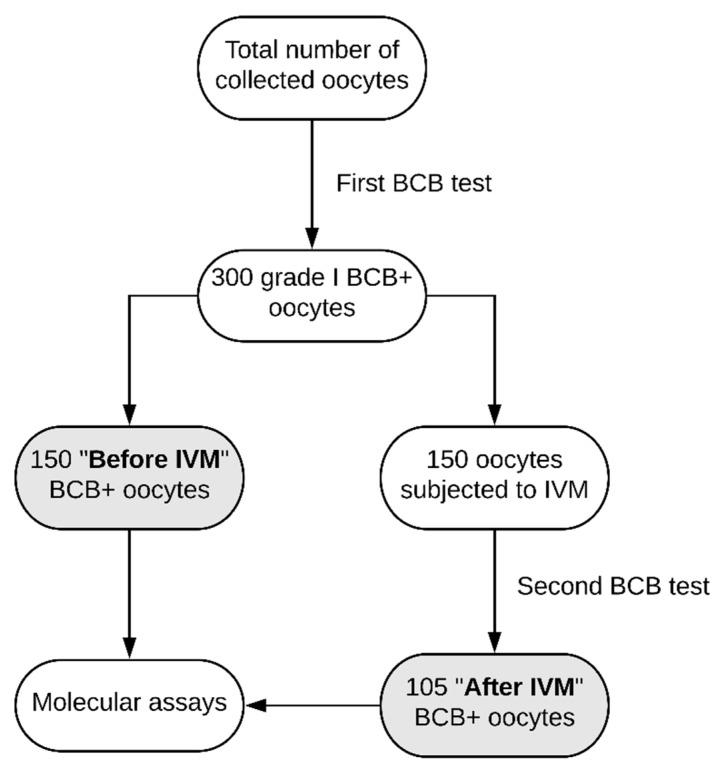
Experimental design, presenting procedures and numbers of oocytes in each stage of selection, applied to every biological replicate.

Brilliant Cresyl Blue (BCB) test was applied to the collected oocytes, which allowed to divide their total number (*n* = 300) into two groups. Half of the oocytes that were graded as BCB-positive (BCB^+^; *n* = 150) were qualified as “Before IVM” group. They were directly subjected to the microarray assay and RT-qPCR. The other half of the oocytes (*n* = 150) were in vitro matured and later analyzed using the same methods, after undergoing a second round of BCB testing and deeming positive (*n* = 105). This group was described as “After IVM”). The total number of oocytes in each group was divided to obtain three biological replicates. The exact processes of selection, maturation and molecular analysis were described in the further parts of the Materials and Methods section (Figure 9).

### 4.2. Animals

In this study, crossbred landrace gilts in a total number of 45, obtained from a local commercial farm were used. Their age ranged from 140–170 days, with 155 days mean. Their mean weight was 100 kg, ranging from 95–120 kg among specimen. The same breeding, housing, and feeding conditions were applied to all of the pigs, based on their age and reproductive status. All experiments were approved by the Poznan University of Medical Sciences Bioethical Committee by resolution 32/2012 (issued 01.06.2012).

### 4.3. Collection of Porcine Ovaries and Cumulus-Oocyte-Complexes (COCs)

At the time of slaughter, the ovaries and reproductive tracts were extracted, with their transport to the laboratory taking no more than 40 min from the moment of specimen death. To ensure material integrity, it was kept at 38 °C in 0.9% NaCl through the course of transit. A bath consisting of 5% fetal bovine serum (FBS; Sigma-Aldrich Co., St. Louis, MO, USA) in PBS was applied to each animal’s ovaries, to ensure optimal conditions for oocyte in vitro maturation and fertilization that followed. A 5 mL syringe and 20-G needle were used to puncture single large follicles (>5 mm) in a sterile Petri dish, which allowed form COC recovery. Modified PBS supplemented with 36 µg/mL pyruvate, 50 µg/mL gentamycin, and 0.5 mg/mL BSA (Sigma-Aldrich, St. Louis, MO, USA) was used for triple washing of the COCs. The COC selection, counting, and morphological evaluation were conducted using a Zeiss Axiovert 35 (Lübeck, Germany) inverted microscope. Only grade I COCs that exhibited homogeneous ooplasm and uniform, compact cumulus cells were qualified for subsequent use, which resulted in a total of 300 grade I oocytes.

Immediately after collection, 10 from 45 ovaries were fixed in Bouin’s solution for 48 h. Subsequently, they were dehydrated and embedded in paraffin blocks according to a routine procedure. Then, they were cut into 3–4 μm thick sections with a semi-automatic rotary microtome (Leica RM 2145, Leica Microsystems, Nussloch, Germany). All ovary paraffin sections were stained with a routine hematoxylin and eosin (H and E) staining method, following the protocol presented (Table 2). Histological sections were evaluated by light microscope and selected pictures were taken with a use of high-resolution scanning technique and Olympus BX61VS microscope scanner (Olympus, Tokyo, Japan).

### 4.4. Assessment of Oocyte Developmental Competence by BCB Test

To assess the quality and maturity of porcine oocytes, the Brilliant Cresyl Blue (BCB) test was used [52]. This assay works by testing the activity of the glucose-6-phosphate (G6PDH) enzyme, which converts BCB stain from blue to colourless. Its activity is largely decreased in oocytes that completed the growth, which causes the inability to remove the stain, resulting in blue oocytes (BCB^+^). Oocytes were washed twice using modified Dulbecco’s Phosphate Buffered Saline (DPBS) to prepare them for BCB staining. The DPBS was commercially supplemented with 0.9 mM calcium, 0.49 mM magnesium, 0.33 mM pyruvate, and 5.5 mM glucose (Sigma-Aldrich, St. Louis, MO, USA), and additionally with 50 IU/mL penicillin, 50 µg/mL streptomycin (Sigma-Aldrich, St. Louis, MO, USA), and 0.4% Bovine Serum Albumin (BSA) (*w*/*v*) (Sigma-Aldrich, St. Louis, MO, USA). The oocytes were afterwards treated with 13 µM BCB (Sigma-Aldrich, St. Louis, MO, USA) diluted in DPBS at 38.5 °C, 5% CO_2_ for 90 min. The oocytes were then, after treatment, transferred to DPBS and washed twice. The oocytes were examined under an inverted microscope during this process, which allowed for their classification as stained blue (BCB^+^) or colourless (BCB^−^). Only BCB^+^ oocytes that were clear of accompanying granulosa cells were used for the following molecular analyses (“Before IVM” group) or IVM followed by second BCB test and molecular analyses (“After IVM” group).

### 4.5. *In Vitro* Maturation of Porcine Cumulus-Oocyte-Complexes (COCs)

The IVM was conducted using oocytes that deemed positive in the first BCB test. Nunclon™Δ 4-well dishes (Thermo Fisher Scientific, Waltham, MA, USA) were used for the oocyte maturation in 500 μL standard porcine IVM culture medium: TCM-199 (tissue culture medium) containing Earle’s salts and l-glutamine (Gibco BRL Life Technologies, Grand Island, NY, USA), supplemented with 2.2 mg/mL sodium bicarbonate (Nacalai Tesque, Inc., Kyoto, Japan), 0.1 mg/mL sodium pyruvate (Sigma-Aldrich, St. Louis, MO, USA), 10 mg/mL BSA (Bovine Serum Albumin) (Sigma-Aldrich, St. Louis, MO, USA), 0.1 mg/mL cysteine (Sigma-Aldrich, St. Louis, MO, USA), 10% (*v*/*v*) filtered porcine follicular fluid, and gonadotropin supplements of 2.5 IU/mL hCG (human Chorionic Gonadotropin) (Ayerst Laboratories, Inc., Philadelphia, PA, USA) and 2.5 IU/mL eCG (equine Chorionic Gonadotropin) (Intervet, Whitby, ON, Canada) final concentrations. For the first 22 h of maturation, the well plates were overlaid with mineral oil and placed in 38 °C, 5% CO_2_. Then, the medium was changed for one without hormone supplementation, with the maturation allowed to proceed in the same conditions for another 22 h. Second BCB test was performed following the maturation, with BCB^+^ oocytes used for subsequent analyses.

### 4.6. Evaluation of Oocytes

At the end of the culture, the part of cultivated oocytes (approximately 10%) was evaluated as a control of meiotic maturation from prophase to second metaphase stage. The oocytes were mounted on slides, fixed with acetic alcohol (1:3, *v*/*v*) for at least 24 h and stained with 1.0% orcein (Sigma-Aldrich, St. Louis, MO, USA), After that, oocytes were examined under a phase contrast microscope, Nikon Eclipse E200, with the stages of meiotic maturation determined (Figure 1). Only experiments with maturation efficiency of ≥ 80% oocytes at second metaphase stage were included in this study.

### 4.7. RNA Extraction from Porcine Oocytes

TRI Reagent (Sigma, St Louis, MO, USA), together with RNeasy MinElute Cleanup Kit (Qiagen, Hilden, Germany) were used for total sample RNA isolation. The final concentration of RNA in resulting batches was determined through the reading optical density at 260 nm, RNA purity was then estimated by the mean of 260/280 nm absorption ratio measurement (>1.8) (NanoDrop spectrophotometer, Thermo Scientific, ALAB, Poland). Bioanalyzer 2100 (Agilent Technologies, Inc., Santa Clara, CA, USA) was used to confirm RNA integrity and quality. The RNA integrity numbers (RINs) obtained had an average of 9.2, varying from 8.5 to 10 between the samples. The final concentration of 100 ng/μL, as well an OD260/OD280 ratio of 1.8/2.0 were achieved in each sample through dilution. 100 ng from was taken from each sample to be used in microarray analyses. The RT-qPCR study used the remaining RNA amount.

### 4.8. Microarray Expression Analysis and Statistics

The Affymetrix procedure was described previously in our papers [6,11,53,54]. Experiments were performed in three replicates. Two round sense cDNA amplification (Ambion® WT Expression Kit, Foster City, CA, USA) were applied to the total pooled RNA from each sample (100 ng). cDNA obtained was used for biotin labelling and fragmentation using Affymetrix GeneChip^®^ WT Terminal Labeling and Hybridization (Affymetrix, Santa Clara, CA, USA). Biotin-labelled fragments of cDNA (5.5 μg) were hybridized to Affymetrix® Porcine Gene 1.1 ST Array Strip (48 °C/20 h). Then, microarrays were washed and stained according to the technical protocol, using Affymetrix GeneAtlas Fluidics Station. The array strips were scanned employing Imaging Station of the GeneAtlas System. The preliminary analysis of the scanned chips was performed using Affymetrix GeneAtlas^TM^ Operating Software. Gene expression quality was examined using software provided control criteria. Downstream data analysis was performed using the obtained CEL files.

BioConductor software (https://www.bioconductor.org/), based on the statistical R programming language, was used to conduct all the analyses. RMA (Robust Multiarray Averaging) was employed to correct background, normalize and summate the raw data. It was a part of the “affy” package of BioConductor. The “oligo” package provided the biological annotation, merging the annotated data frame object with a normalized data set, resulting in a complete gene data table. Moderated *t*-statistics from the empirical Bayes method were used to establish the statistical significance of the analyzed genes. Benjamini and Hochberg’s false discovery rate was used to correct the obtained *p*-value. The significantly changed genes were selected if they exhibited fold change higher than |2| and *p*-value < 0.05.

DAVID (Database for Annotation, Visualization and Integrated Discovery) was used to functionally annotate and cluster the genes that were considered differentially expressed. Up- or down-regulated gene symbols belonging to the compared groups were uploaded to DAVID by “RDAVIDWebService” BioConductor package. For further analysis, we have chosen the enriched GO terms which had at least 5 genes and *p*-value (Benjamini) lower than 0.05. The enriched GO terms were subjected to hierarchical clusterization algorithm and presented as heatmaps.

Subsequently, we analyzed the relationship between the genes belonging to chosen GO terms using the GOplot package [55]. The GOPlot package had calculated the Z-score: up-regulated gene number, with the number of down-regulated genes subtracted, divided by the square root of the count. This information allowed estimating the changing course of each gene-ontology term.

Among genes that build chosen GO terms, we have chosen 10 most up and 10 most downregulated. Interactions between chosen differentially expressed genes/proteins belonging to the analyzed gene ontologies were investigated with the use of STRING10 (Search Tool for the Retrieval of Interacting Genes) software (https://string-db.org/). Interaction prediction was performed using the list of gene names as a query. Co-occurrences of genes/proteins in scientific texts (text mining), co-expression and experimentally observed interactions were used as a search criteria. The results of that search yielded a gene/protein interaction network, with the intensity of the edges reflecting the strength of the interaction score. Based on previously uploaded gene sets, STRING also allowed us to conduct functional enrichment of GO terms.

Finally, the functional interactions between genes that belong to the chosen GO BP terms were investigated by REACTOME FIViz application of the Cytoscape 3.6.0 software (https://cytoscape.org/; http://apps.cytoscape.org/apps/reactomefiplugin). This app serves to locate pathways and network patterns that are connected to cancer and other disease types. It allows to conduct pathway enrichment analysis for a specified gene set, visualises found pathways with the use of manually laid-out pathway diagrams, as well as to investigate the functional relations between the genes of those pathways, through accessing the Reactome database. Additionally, it can access a highly reliable, pathway-based, manually curated pathway based protein functional interaction network that covers over 60% of human proteins, the Reactome FI (functional interaction) networks.

### 4.9. Real-time Quantitative Polymerase Chain Reaction (RT-qPCR) Analysis

Total RNA was isolated from oocytes before and/or after IVM. The RNA samples were stored in liquid nitrogen after re-suspension in 20 µL of RNase-free water. DNase I treatment was applied to the RNA samples before they have been reverse-transcribed (RT) into cDNA. LightCycler real-time PCR detection system (Roche Diagnostics GmbH, Mannheim, Germany) was used to perform the RQ-PCR reactions. SYBR^®^ Green I was employed as a detection dye, with target cDNA quantified using the relative quantification method. G*lyceraldehyde-3-phosphate dehydrogenase* (*GAPDH*) served as an internal standard when measuring the abundance of *ID2*, *VEGFA*, *BTG2*, *ESR1*, *CCND2*, *EDNRA*, *ANGPTL4*, *TGFBR3*, *GJA1*, *LAMA2*, *KIT*, *TPM1*, *VCP*, *GRID2*, *MEF2C*, *RPS3A*, *PLD1*, *BTG3*, *CD47*, *MITF* transcripts in the analyzed samples. 2 µL of cDNA solution was mixed with 18 µL of QuantiTect^®^ SYBR^®^ Green PCR (Master Mix Qiagen GmbH, Hilden, Germany) and primers (Table 3) and used in the amplification process. To provide a negative control for following PCR, one RNA repeat from each sample was processed without the RT-reaction.

Expression levels of specific oocyte mRNAs were calculated in relation to *PBGD* and *ACTB* to quantify genes specific for the oocyte. An additional housekeeping gene, *18S rRNA*, was used as an internal standard, to ensure result integrity. Its role was to demonstrate that, in analyzed samples, *PBGD* and *ACTB* mRNAs were not differentially expressed. *18S rRNA* was previously determined appropriate as a housekeeping gene in quantitative PCR studies.

## Figures and Tables

**Table 1:** Gene symbols, fold changes in expression, and corrected p values of studied genes. All fold changes were calculated as [after in vitro maturation transcript levels/before in vitro maturation transcript levels], with “before IVM” group serving as a point of reference.

Gene	Fold Change	*p*-Value
*ID2*	0.062979704	4.74E-05
*VEGFA*	0.069689389	0.001912689
*BTG2*	0.074386393	9.55E-05
*ESR1*	0.081629841	0.000522187
*CCND2*	0.121809064	0.000178804
*EDNRA*	0.166939028	0.00185422
*ANGPTL4*	0.183631311	0.000513422
*TGFBR3*	0.196522244	0.000405979
*GJA1*	0.206907347	0.000107676
*LAMA2*	0.219665855	0.000794627
*KIT*	0.430444215	0.00255635
*TPM1*	0.433963109	0.001632742
*VCP*	0.435612412	0.007402292
*GRID2*	0.444288529	0.008594981
*MEF2C*	0.453593212	0.00396401
*RPS3A*	0.462721116	0.002620209
*PLD1*	0.468341554	0.011044722
*BTG3*	0.485731167	0.040345869
*CD47*	0.486714062	0.009288999
*MITF*	0.491659656	0.006329736

**Table 2:** The stages of H and E staining protocol, detailing the reagents and reaction times.

Steps	Solution	Duration
Deparaffinization and rehydration	Xylene I	10 min
	Xylene II	5 min
	Absolute alcohol	5 min
	90% alcohol	5 min
	70% alcohol	5 min
	Water wash	10 min
Staining	Hematoxylin	20 min
	Water wash	8 min
	Eosin	20 min
	Water wash	8 min
Dehydration	70% alcohol	5 min
	90% alcohol	5 min
	Absolute alcohol	5 min
	Xylene I	5 min
	Xylene II	10 min

**Table 3:** Primers Oligonucleotide sequences of primers used for RT-qPCR analysis.

Gene	Gene Accession Number	Primer Sequence (5′-3′)	Product Size (bp)
*ID2*	NM_001037965.1	CCAGTGAGGTCCGTTAGGAA	243
		GACAATAGTGGGGTGCGAGT	
*VEGFA*	NM_214084.1	CTACCTCCACCATGCCAAGT	232
		ACACTCCAGACCTTCGTCGT	
*BTG2*	NM_001097505.2	TGGTTTCCTGAAAAGCCATC	150
		GGACACTTCATAGGGGTCCA	
*ESR1*	NM_214220.1	AGCACCCTGAAGTCTCTGGA	160
		TGTGCCTGAAGTGAGACAGG	
*CCND2*	NM_214088.1	CGTCCAAGCTCAAAGAGACC	169
		CGAAGAATGTGCTCGATGAA	
*EDNRA*	NM_214229.1	CACCACATTTCGTGGAACAG	205
		AATGATCCTGAGCAGGGTTG	
*ANGPTL4*	NM_214229.1	TGCCAAGAGCTGTTTGAAGA	152
		TTAAAGTCCACCGAGCCATC	
*TGFBR3*	NM_214272.1	TCATCTCCCCGTACTCGAAC	219
		TCTTGGTACACAGCGTGAGC	
*GJA1*	NM_001244212.1	CACCAGGTGGACTGTTTCCT	151
		TCTTTCCCTTCACACGATCC	
*LAMA2*	XM_021084463.1	CTCCAGGCTATTCTGGCTTG	153
		AGGTTTCAGGGTCACACAGG	
*KIT*	NM_001044525.1	TCAGGGAGAACAGCCAGACT	170
		GGTGGTTGTGACATTTGCAG	
*TPM1*	NM_001097483.2	CTCTGAACAGACGCATCCAA	243
		TACGGGCCACCTCTTCATAC	
*VCP*	NM_214280.1	ACCCTCCAAGGGAGTGCTAT	223
		GGCAATTGAATCCAGCTCAT	
*GRID2*	XM_021100736.1	GTCCCATCGAAAGAGGATGA	176
		TCACTGATTTGCTCCAGTGC	
*MEF2C*	NM_001044540.1	GCCCTGAGTCTGAGGACAAG	163
		AGTGAGCTGACAGGGTTGCT	
*RPS3A*	NM_001137619.1	CCGGAAGAAGATGATGGAAA	179
		CAAACTTGGGCTTCTTCAGC	
*PLD1*	NM_001244589.1	TTCTGGACCCAGTGAAGACC	229
		GAACCCACGGATCTTTTTCA	
*BTG3*	NM_001097517.1	TACAGATGCATTCGGGTCAA	153
		CTCTCCATACCGACAGCACA	
*CD47*	NM_213982.1	TGGAGCCATTCTTTTCATCC	191
		GTCCAACCACAGAGAGCACA	
*MITF*	NM_001038001.1	GCCAATCGGCATTTGTTACT	236
		CCGAGGTTGTTGTTGAAGGT	

## References

[1] Hunt P.A., Hassold T.J. (2008). Human female meiosis: What makes a good egg go bad?. Trends Genet..

[2] Viswanathan S.R., Mermel C.H., Lu J., Lu C.-W., Golub T.R., Daley G.Q. (2009). microRNA Expression during Trophectoderm Specification. PLoS ONE.

[3] Sinha P.B., Tesfaye D., Rings F., Hossien M., Hoelker M., Held E., Neuhoff C., Tholen E., Schellander K., Salilew-Wondim D. (2017). MicroRNA-130b is involved in bovine granulosa and cumulus cells function, oocyte maturation and blastocyst formation. J. Ovarian Res..

[4] Kempisty B., Ziółkowska A., Ciesiółka S., Piotrowska H., Antosik P., Bukowska D., Nowicki M., Brüssow K.P., Zabel M. (2014). Study on connexin gene and protein expression and cellular distribution in relation to real-time proliferation of porcine granulosa cells. J. Biol. Regul. Homeost. Agents.

[5] Diaz F.J., Wigglesworth K., Eppig J.J. (2007). Oocytes are required for the preantral granulosa cell to cumulus cell transition in mice. Dev. Biol..

[6] Rybska M., Knap S., Jankowski M., Jeseta M., Bukowska D., Antosik P., Nowicki M., Zabel M., Kempisty B., Jaśkowski J.M. (2018). Cytoplasmic and nuclear maturation of oocytes in mammals–living in the shadow of cells developmental capability. Med. J. Cell Biol..

[7] Gilchrist R., Ritter L., Armstrong D. (2004). Oocyte–somatic cell interactions during follicle development in mammals. Anim. Reprod. Sci..

[8] Li R., Norman R.J., Armstrong D.T., Gilchrist R.B. (2000). Oocyte-secreted factor(s) determine functional differences between bovine mural granulosa cells and cumulus cells. Biol. Reprod..

[9] Macaulay A.D., Gilbert I., Caballero J., Barreto R., Fournier E., Tossou P., Sirard M.-A., Clarke H.J., Khandjian É.W., Richard F.J. (2014). The Gametic Synapse: RNA Transfer to the Bovine Oocyte^1^. Biol. Reprod..

[10] Biase F.H., Kimble K.M. (2018). Functional signaling and gene regulatory networks between the oocyte and the surrounding cumulus cells. BMC Genomics.

[11] Nawrocki M.J., Celichowski P., Budna J., Bryja A., Kranc W., Ciesiółka S., Borys S., Knap S., Jeseta M., Khozmi R. (2017). The blood vessels development, morphogenesis and blood circulation are three ontologic groups highly up-regulated in porcine oocytes before in vitro maturation. Adv. Cell Biol..

[12] Budna J., Bryja A., Celichowski P., Kranc W., Ciesiółka S., Borys S., Rybska M., Kolecka-Bednarczyk A., Jeseta M., Bukowska D. (2017). “Bone Development” Is an Ontology Group Upregulated in Porcine Oocytes Before In Vitro Maturation: A Microarray Approach. DNA Cell Biol..

[13] Kordus R.J., LaVoie H.A. (2017). Granulosa cell biomarkers to predict pregnancy in ART: Pieces to solve the puzzle. Reproduction.

[14] Rybska M., Knap S., Jankowski M., Jeseta M., Bukowska D., Antosik P., Nowicki M., Zabel M., Kempisty B., Jaśkowski J.M. (2018). Characteristic of factors influencing the proper course of folliculogenesis in mammals. Med. J. Cell Biol..

[15] Barrett S.L., Albertini D.F. (2010). Cumulus cell contact during oocyte maturation in mice regulates meiotic spindle positioning and enhances developmental competence. J. Assist. Reprod. Genet..

[16] Komatsu K., Masubuchi S. (2018). Mouse oocytes connect with granulosa cells by fusing with cell membranes and form a large complex during follicle development. Biol. Reprod..

[17] Chachuła A., Kranc W., Budna J., Bryja A., Ciesiólka S., Wojtanowicz-Markiewicz K., Piotrowska H., Bukowska D., Krajecki M., Antosik P. (2016). The differentiation of mammalian ovarian granulosa cells living in the shadow of cellular developmental capacity. J. Biol. Regul. Homeost. Agents.

[18] Budna J., Celichowski P., Karimi P., Kranc W., Bryja A., Ciesiółka S., Rybska M., Borys S., Jeseta M., Bukowska D. (2017). Does Porcine Oocytes Maturation in Vitro is Regulated by Genes Involved in Transforming Growth Factor B Receptor Signaling Pathway?. Adv. Cell Biol..

[19] Hutt K.J., Albertini D.F. (2007). An oocentric view of folliculogenesis and embryogenesis. Reprod. Biomed. Online.

[20] Dias F.C.F., Khan M.I.R., Sirard M.A., Adams G.P., Singh J. (2018). Transcriptome analysis of granulosa cells after conventional vs long FSH-induced superstimulation in cattle. BMC Genomics.

[21] Zheng W., Liu K. (2012). Maternal Control of Mouse Preimplantation Development. Results Probl. Cell Differ..

[22] Kranc W., Budna J., Chachuła A., Borys S., Bryja A., Rybska M., Ciesiółka S., Sumelka E., Jeseta M., Brüssow K.P. (2017). “Cell Migration” Is the Ontology Group Differentially Expressed in Porcine Oocytes Before and After In Vitro Maturation: A Microarray Approach. DNA Cell Biol..

[23] Kempisty B., Ziółkowska A., Piotrowska H., Zawierucha P., Antosik P., Bukowska D., Ciesiółka S., Jaśkowski J.M., Brüssow K.P., Nowicki M., Zabel M. (2013). Real-time proliferation of porcine cumulus cells is related to the protein levels and cellular distribution of Cdk4 and Cx43. Theriogenology.

[24] Borup R., Thuesen L.L., Andersen C.Y., Nyboe-Andersen A., Ziebe S., Winther O., Grøndahl M.L. (2016). Competence Classification of Cumulus and Granulosa Cell Transcriptome in Embryos Matched by Morphology and Female Age. PLoS ONE.

[25] Borys S., Khozmi R., Kranc W., Bryja A., Dyszkiewicz-Konwińska M., Jeseta M., Kempisty B. (2017). Recent findings of the types of programmed cell death. Adv. Cell Biol..

[26] Artini P.G., Tatone C., Sperduti S., D’Aurora M., Franchi S., Di Emidio G., Ciriminna R., Vento M., Di Pietro C., Stuppia L. (2017). Cumulus cells surrounding oocytes with high developmental competence exhibit down-regulation of phosphoinositol 1, 3 kinase/protein kinase B (PI3K/AKT) signalling genes involved in proliferation and survival. Hum. Reprod..

[27] Massari M.E., Murre C. (2000). Helix-loop-helix proteins: Regulators of transcription in eucaryotic organisms. Mol. Cell. Biol..

[28] Budna J., Chachuła A., Kaźmierczak D., Rybska M., Ciesiółka S., Bryja A., Kranc W., Borys S., Żok A., Bukowska D. (2017). Morphogenesis-related gene-expression profile in porcine oocytes before and after in vitro maturation. Zygote.

[29] Guo L., Lan J., Lin Y., Guo P., Nie Q., Mao Q., Ren L., Qiu Y. (2013). Hypoxia/ischemia up-regulates Id2 expression in neuronal cells in vivo and in vitro. Neurosci. Lett..

[30] Hazzard T.M., Xu F., Stouffer R.L. (2002). Injection of soluble vascular endothelial growth factor receptor 1 into the preovulatory follicle disrupts ovulation and subsequent luteal function in rhesus monkeys. Biol. Reprod..

[31] Trau H.A., Brännström M., Curry T.E., Duffy D.M. (2016). Prostaglandin E2 and vascular endothelial growth factor A mediate angiogenesis of human ovarian follicular endothelial cells. Hum. Reprod..

[32] Kranc W., Celichowski P., Budna J., Khozmi R., Bryja A., Ciesiółka S., Rybska M., Borys S., Jeseta M., Bukowska D. (2017). Positive Regulation Of Macromolecule Metabolic Process Belongs To The Main Mechanisms Crucial For Porcine Ooocytes Maturation. Adv. Cell Biol..

[33] Anchordoquy J.M., Anchordoquy J.P., Testa J.A., Sirini M.Á., Furnus C.C. (2015). Influence of vascular endothelial growth factor and Cysteamine on in vitro bovine oocyte maturation and subsequent embryo development. Cell Biol. Int..

[34] Celichowski P., Nawrocki M.J.M.J., Dyszkiewicz-Konwińska M., Jankowski M., Budna J., Bryja A., Kranc W., Borys S., Knap S., Ciesiółka S. (2018). “Positive Regulation of RNA Metabolic Process” Ontology Group Highly Regulated in Porcine Oocytes Matured In Vitro: A Microarray Approach. Biomed Res. Int..

[35] Tirone F. (2001). The gene PC3TIS21/BTG2, prototype member of the PC3/BTG/TOB family: Regulator in control of cell growth, differentiation, and DNA repair?. J. Cell. Physiol..

[36] Goldenberg R.L., Vaitukaitis J.L., Ross G.T. (1972). Estrogen and Follicle Stimulating Hormone Interactions on Follicle Growth in Rats. Endocrinology.

[37] Al-Edani T., Assou S., Ferrières A., Bringer Deutsch S., Gala A., Lecellier C.-H., Aït-Ahmed O., Hamamah S. (2014). Female aging alters expression of human cumulus cells genes that are essential for oocyte quality. Biomed Res. Int..

[38] Robker R.L., Richards J.S. (1998). Hormonal control of the cell cycle in ovarian cells: Proliferation versus differentiation. Biol. Reprod..

[39] Van Montfoort A.P.A., Geraedts J.P.M., Dumoulin J.C.M., Stassen A.P.M., Evers J.L.H., Ayoubi T.A.Y. (2008). Differential gene expression in cumulus cells as a prognostic indicator of embryo viability: A microarray analysis. Mol. Hum. Reprod..

[40] Bigham A.W., Julian C.G., Wilson M.J., Vargas E., Browne V.A., Shriver M.D., Moore L.G. (2014). Maternal PRKAA1 and EDNRA genotypes are associated with birth weight, and PRKAA1 with uterine artery diameter and metabolic homeostasis at high altitude. Physiol. Genomics.

[41] Kawamura K., Ye Y., Liang C.G., Kawamura N., Gelpke M.S., Rauch R., Tanaka T., Hsueh A.J.W. (2009). Paracrine regulation of the resumption of oocyte meiosis by endothelin-1. Dev. Biol..

[42] Wang H.X., Tong D., El-Gehani F., Tekpetey F.R., Kidder G.M. (2009). Connexin expression and gap junctional coupling in human cumulus cells: Contribution to embryo quality. J. Cell. Mol. Med..

[43] Li S.-H., Lin M.-H., Hwu Y.-M., Lu C.-H., Yeh L.-Y., Chen Y.-J., Lee R.K.-K. (2015). Correlation of cumulus gene expression of GJA1, PRSS35, PTX3, and SERPINE2 with oocyte maturation, fertilization, and embryo development. Reprod. Biol. Endocrinol..

[44] DeLaughter D.M., Clark C.R., Christodoulou D.C., Seidman C.E., Baldwin H.S., Seidman J.G., Barnett J.V. (2016). Transcriptional Profiling of Cultured, Embryonic Epicardial Cells Identifies Novel Genes and Signaling Pathways Regulated by TGFβR3 In Vitro. PLoS ONE.

[45] Sharma S.M., Sif S., Ostrowski M.C., Sankar U. (2009). Defective co-activator recruitment in osteoclasts from *microphthalmia-oak ridge* mutant mice. J. Cell. Physiol..

[46] Mathias M.D., Sockolosky J.T., Chang A.Y., Tan K.S., Liu C., Garcia K.C., Scheinberg D.A. (2017). CD47 blockade enhances therapeutic activity of TCR mimic antibodies to ultra-low density cancer epitopes. Leukemia.

[47] Lv C., Wang H., Tong Y., Yin H., Wang D., Yan Z., Liang Y., Wu D., Su Q. (2018). The function of BTG3 in colorectal cancer cells and its possible signaling pathway. J. Cancer Res. Clin. Oncol..

[48] Garrido J.L., Wheeler D., Vega L.L., Friedman P.A., Romero G. (2009). Role of Phospholipase D in Parathyroid Hormone Type 1 Receptor Signaling and Trafficking. Mol. Endocrinol..

[49] Tang Y., He Y., Li C., Mu W., Zou Y., Liu C., Qian S., Zhang F., Pan J., Wang Y. (2018). RPS3A positively regulates the mitochondrial function of human periaortic adipose tissue and is associated with coronary artery diseases. Cell Discov..

[50] Ali Z., Zulfiqar S., Klar J., Wikström J., Ullah F., Khan A., Abdullah U., Baig S., Dahl N. (2017). Homozygous GRID2 missense mutation predicts a shift in the D-serine binding domain of GluD2 in a case with generalized brain atrophy and unusual clinical features. BMC Med. Genet..

[51] Bastola P., Neums L., Schoenen F.J., Chien J. (2016). VCP inhibitors induce endoplasmic reticulum stress, cause cell cycle arrest, trigger caspase-mediated cell death and synergistically kill ovarian cancer cells in combination with Salubrinal. Mol. Oncol..

[52] Roca J., Martinez E., Vazquez J.M., Lucas X. (1998). Selection of immature pig oocytes for homologous in vitro penetration assays with the brilliant cresyl blue test. Reprod. Fertil. Dev..

[53] Nawrocki M.J., Budna J., Celichowski P., Khozmi R., Bryja A., Kranc W., Borys S., Ciesiółka S., Knap S., Jeseta M. (2017). Analysis of fructose and mannose–regulatory peptides signaling pathway in porcine epithelial oviductal cells (OECs) primary cultured long-term in vitro. Adv. Cell Biol..

[54] Kranc W., Jankowski M., Budna J., Celichowski P., Khozmi R., Bryja A., Borys S., Dyszkiewicz-Konwińska M., Jeseta M., Magas M. (2018). Amino acids metabolism and degradation is regulated during porcine oviductal epithelial cells (OECs) primary culture in vitro—A signaling pathways activation approach. Med. J. Cell Biol..

[55] Walter W., Sánchez-Cabo F., Ricote M. (2015). GOplot: An R package for visually combining expression data with functional analysis. Bioinformatics.

